# Risk Factors for Frequent Readmissions and Barriers to Transplantation in Patients with Cirrhosis

**DOI:** 10.1371/journal.pone.0055140

**Published:** 2013-01-28

**Authors:** Swaytha Ganesh, Shari S. Rogal, Dhiraj Yadav, Abhinav Humar, Jaideep Behari

**Affiliations:** 1 Department of Medicine, Division of Gastroenterology, Hepatology, and Nutrition, University of Pittsburgh, Pittsburgh, Pennsylvania, United States of America; 2 Department of Surgery, Division of Transplantation, University of Pittsburgh, Pittsburgh, Pennsylvania, United States of America; University of Navarra School of Medicine and Center for Applied Medical Research (CIMA), Spain

## Abstract

**Background:**

Hospital readmission rate is receiving increasing regulatory scrutiny. Patients with cirrhosis have high hospital readmissions rates but the relationship between frequent readmissions and barriers to transplantation remains unexplored. The goal of this study was to determine risk factors for frequent readmissions among patients with cirrhosis and identify barriers to transplantation in this population.

**Methods:**

We retrospectively reviewed medical records of 587 patients with a confirmed diagnosis of cirrhosis admitted to a large tertiary care center between May 1, 2008 and May 1, 2009. Demographics, clinical factors, and outcomes were recorded. Multivariate logistic regression was performed to identify risk factors for high readmission rates. Transplant-related factors were assessed for patients in the high readmission group.

**Results:**

The 587 patients included in the study had 1557 admissions during the study period. A subset of 87 (15%) patients with 5 or more admissions accounted for 672 (43%) admissions. The factors associated with frequent admissions were non-white race (OR = 2.45, p = 0.01), diabetes (OR = 2.04, p = 0.01), higher Model for End-Stage Liver Disease (MELD) score (OR = 35.10, p<0.0001 for MELD>30) and younger age (OR = 0.98, p = 0.02). Among the 87 patients with ≥5 admissions, only 14 (16%) underwent liver transplantation during the study period. Substance abuse, medical co-morbidities, and low (<15) MELD scores were barriers to transplantation in this group.

**Conclusions:**

A small group of patients with cirrhosis account for a disproportionately high number of hospital admissions. Interventions targeting this high-risk group may decrease frequent hospital readmissions and increase access to transplantation.

## Introduction

The high frequency and cost of readmissions are important factors underlying the increase in healthcare costs in the United States (US). Recent studies have demonstrated that 20% of Medicare beneficiaries are re-hospitalized within 30 days and 34% within 90 days and the majority have medical rather than surgical indications for admission [1]. The estimated cost of unplanned readmissions was $17.4 billion in 2004, which comprised 20% of Medicare’s hospital payments [1], [2]. Therefore, readmission rates have been proposed as a national quality indicator and the search for modifiable risk factors for readmissions is considered critical to limiting healthcare spending [3].

Cirrhosis leads to over 150,000 hospitalizations at an annual cost of nearly $4 billion in the US [4]–[6]. A recent study found that the readmission rate among patients with cirrhosis-related complications was 20% at 30 days [7]. Another study found a 30-day readmission rate of 37% among patients with decompensated cirrhosis with an overall rate of 3 hospitalizations per person-year [8]. Since the prevalence of end-stage liver disease is expected to increase due to the increasing burden of hepatitis C- and non-alcoholic fatty liver disease, hospitalizations from complications of cirrhosis and the associated risk of readmissions are also likely to increase [9]–[12].

Attempts to decrease frequency of readmissions in patients with cirrhosis are likely to be most cost-effective if they are directed towards patients at the highest risk of readmissions. Thus, we sought to identify risk factors for frequent admissions in cirrhosis patients that may be amenable to targeted interventions. Furthermore, since liver transplantation is the only definitive therapy available for decompensated cirrhosis, we hypothesized that patients with frequent readmissions have significant barriers to liver transplantation. We show here that a relatively small group of cirrhosis patients accounts for a surprisingly large number of hospital admissions and identify potential barriers to transplantation in this cohort.

## Methods

We queried electronic medical records at the University of Pittsburgh Medical Center and identified 1,802 patients admitted with the ICD-9 code 571 (chronic liver disease and cirrhosis) between May 1, 2008 and May 1, 2009. After review of each patient’s chart by an experienced hepatologist, 823 patients were excluded because they did not have cirrhosis based on imaging studies, liver biopsy, or clinical documentation of a cirrhosis-related complication. To ensure adequate time for follow-up and opportunity for frequent readmissions as well as to homogenize the length of follow-up, 211 patients who did not have at least 90 days of follow-up were also excluded. Patients with total parenteral nutrition (TPN)-induced liver failure (N = 11), fulminant liver failure (N = 44), metastatic or neuroendocrine tumors of the liver (N = 43), prior liver transplant (N = 24), and death or transplant during the index admission (N = 59) were also excluded. Thus, a total of 587 patients with confirmed cirrhosis admitted during the study period were included in the final analysis.

The mean number of admissions per person-year in this cohort was 4.25, based on which we divided the patients into the following two groups: those with 4 or fewer admissions (including the initial admission) and those with 5 or more admissions (the “high-readmission” group). Baseline characteristics were calculated for both groups. The etiology of cirrhosis was categorized as due to alcohol, viral hepatitis (with or without alcohol), or other and then as alcohol-related or non-alcohol related. The reason for initial admission was categorized as cirrhosis-related vs. non-cirrhosis related. Among patients with cirrhosis-related admissions, reasons for readmission were categorized as fluid-related (including ascites, hydrothorax, or renal failure related to liver disease), encephalopathy-related and bleeding-related (esophageal or gastric variceal bleeding). The fluid-related problems were collapsed to allow sufficient numbers for inclusion in multivariate regression models. Length of follow-up was calculated for each person from the date of discharge after the index admission until the end of the study period or death. T-tests were used to compare continuous variables, and Chi-square test for categorical variables. For non-normal data, medians and interquartile ranges were calculated and Wilcoxon-Rank Sum test was used for comparisons of medians. Fisher’s exact test was used to compare categorical variables with expected cell counts of less than 5.

A logistic regression model was created to compare patients with 5 or more admissions to those with <5 admissions using variables from baseline characteristics with p<0.2 in comparisons and using dummy variables for a priori categories of MELD scores. Race was coded as white vs. non-white for the purposes of logistic regression. The reason for index admission was coded into dummy variables including encephalopathy and volume-related admissions. Length of follow up was included in the model to account for differences, and this variable accounted for time of death. Odds ratios, p values, and 95% confidence intervals were calculated for each variable, and variance inflation factors were checked in the final model to ensure there was no multicollinearity. To create the most parsimonious model, variables were removed in a backward elimination fashion until only significant predictors remained (p<0.05). To control for bias introduced by excluding those with <90 days of follow-up, we also tested a similar model by including these individuals in the analysis. To check the assumptions of the model and avoid picking a cut-point for number of admissions, a linear regression model was created. All covariates were included in the initial model, and a stepwise backwards elimination was completed to leave a parsimonious model with covariates with p<0.1 remaining.

For the 87 patients in the high-readmission group, change in MELD score from the first to the last admission was calculated, and this was used to calculate change in MELD per person per month of follow up. To identify the barriers to liver transplant in this group, we reviewed their charts to ascertain dates of transplant referral, listing, and (if applicable) the reason for their not being referred or listed. The mean number of admissions between referral and listing was calculated for the patients who were evaluated for liver transplantation. The mean time between referral and listing was also calculated for patients that were listed for transplant.

### Ethics Statement

Prior approval for this study was obtained from the University of Pittsburgh Medical Center Quality Improvement Committee. The study was conducted as a consent-waived quality improvement project with adherence to all ethical and patient confidentiality standards of our institution.

## Results

**Figure 1:**
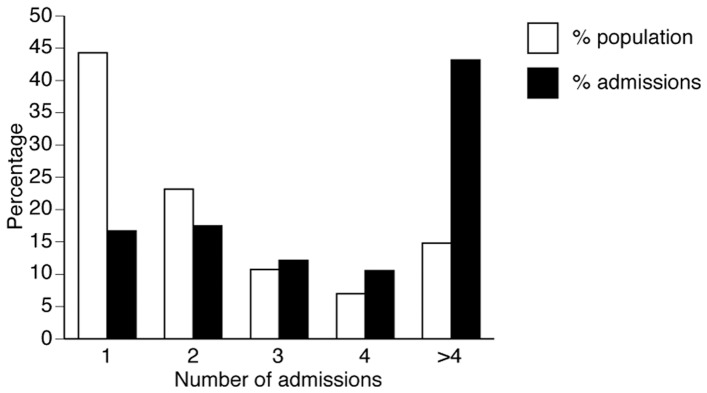
A small group of cirrhosis patients accounts for a dispropionate percentage of hospital admissions. The percentage of patients with one or more admissions during the study period is shown.

The 587 cirrhosis patients included in the study had a total of 1,557 admissions during the one-year study period. The mean number of admissions per person-year was 4.25±3.8, with a mean absolute number of admissions of 2.7±2.6. A group of 87 patients with 5 or more admissions made up 15% of the study group but contributed 43% (n = 672) of the admissions (high-readmission group; Figure 1).

Patients in the high-readmission group were younger and more likely to be non-white (Table 1). They were also more likely to have diabetes, higher baseline MELD scores, and alcohol-related cirrhosis. The mean length of follow up was 232 days in the low-readmission (4 or less total admission) group vs. 281 days in the high-readmission group. The follow-up time difference between the groups was 1.5 months, compared to a median time of follow-up of 9 months. Marital and family situations were similar between the groups. The reason for initial admission in the high-readmission group was more likely to be related to a complication of cirrhosis and more likely to be specifically related to hepatic encephalopathy or volume-related issue (ascites, hydrothorax, and/or renal failure). The high-readmission group had longer initial length of stay and was more likely to die or undergo liver transplantation during the study period.

**Table 1:** Comparison of baseline characteristics based on number of admissions.

Characteristics	Less than 5 total admissions (N = 500)	5 or more total admissions (N = 87)	P value
Age*	56.7±12.6	53.1±12.0	0.01
Male gender N (%)	278 (56)	39 (45)	0.97
Race N (%)			0.02Ŧ
Caucasian	438 (88)	69 (79)	
African-American	44 (9)	15 (17)	
Other	4 (1)	2 (2)	
Married N (%)	270 (54)	41 (47)	0.28
Diabetes	52 (10)	35 (40)	0.03
BMI*	26.5±5.3	27.3±6.2	0.43
Etiology of Liver Disease			0.24
Alcohol	121 (24)	28 (32)	
Viral +/− Alcohol	176 (35)	25 (29)	
Other	203 (41)	34 (39)	
Alcohol-related etiology	139 (28)	34 (39)	0.045
MELD score N (%)			<0.0001
<15	276 (55)	12 (14)	
15–20	141 (28)	44 (51)	
21–30	79 (16)	27 (31)	
31–40	4 (1)	4 (5)	
MELD score index admission*	14.4±6.0	19.1±4.8	<0.0001
Blood Type			0.39 Ŧ
O	180 (36)	35 (40)	
A	155 (31)	31 (36)	
B	46 (9)	10 (11)	
AB	7 (1)	4 (5)	
Initial length of stay (days)* ^ +^	5.0±5.13.8 (2.0, 6.0)	5.4±4.54.1 (2.7, 7.2)	0.430.10
Non-cirrhosis-related index admission	248 (50)	22 (26)	<0.0001
Encephalopathy-related index admission	74 (15)	21 (24)	0.04
Volume-related index admission**	91 (18)	31 (36)	0.0004
Bleeding-related index admission	38 (8)	6 (7)	0.99
Length of follow up (days)*	232+82	281±70	<0.0001
Death during study N (%)	28 (6)	14 (16)	0.001
Transplant during study N (%)	30 (6)	14 (16)	0.002

* Mean ± sd, p value calculated using t-test.

Ŧ Fisher’s Exact test used, numbers do not sum to 100% given missing values.

** Volume-related index admission = renal failure, ascites, or hydrothorax.

In the final logistic regression model (Table 2), high readmissions rate was associated with younger age and non-white race (OR = 2.45, 95%CI = 1.19, 4.94). Diabetes also increased the odds of admissions (OR = 2.04, 95%CI = 1.18, 3.54). The high-readmission group had significantly higher MELD scores. The mean change in MELD over the study period was 0.87±4.75 points with a median change of 0 (range = −8 to 18, IQR = −2,3). The change per month was 0.14±0.78, with a median change of 0 (range = −2 to 2.6, IQR = −0.3 to 0.34). A logistic regression model was made including those with <90 day follow-up, and the median follow-up time was expectedly more disparate between those with and without at least 5 admissions (median 286 vs 174 days). By including patients with <90 days of follow-up, only 3 additional patients were added to the high-readmission group and the final model was similar to that obtained by excluding patients with short follow-up time. However, age and cirrhosis-related index admission became non-significant, and alcohol-related etiology of liver disease became a significant factor.

**Table 2:** Final logistic regression model comparing characteristics of those with 5 or more admissions to those with less admission.

Variable	OR	95% CI	P
Age	0.98	0.95, 0.996	0.02
Non-white race	2.45	1.19, 4.94	0.01
Diabetes	2.04	1.18, 3.54	0.01
MELD score (vs. those 14 or less)			
15–20	6.55	3.35, 13.68	<0.0001
21–30	8.58	4.12, 18.88	<0.0001
31–40	35.10	6.82, 186.29	<0.0001
Length follow up*	1.01	1.005,1.01	<0.0001

* Follow up time was from the index discharge to the end of the study period or to time of death.

**Figure 2:**
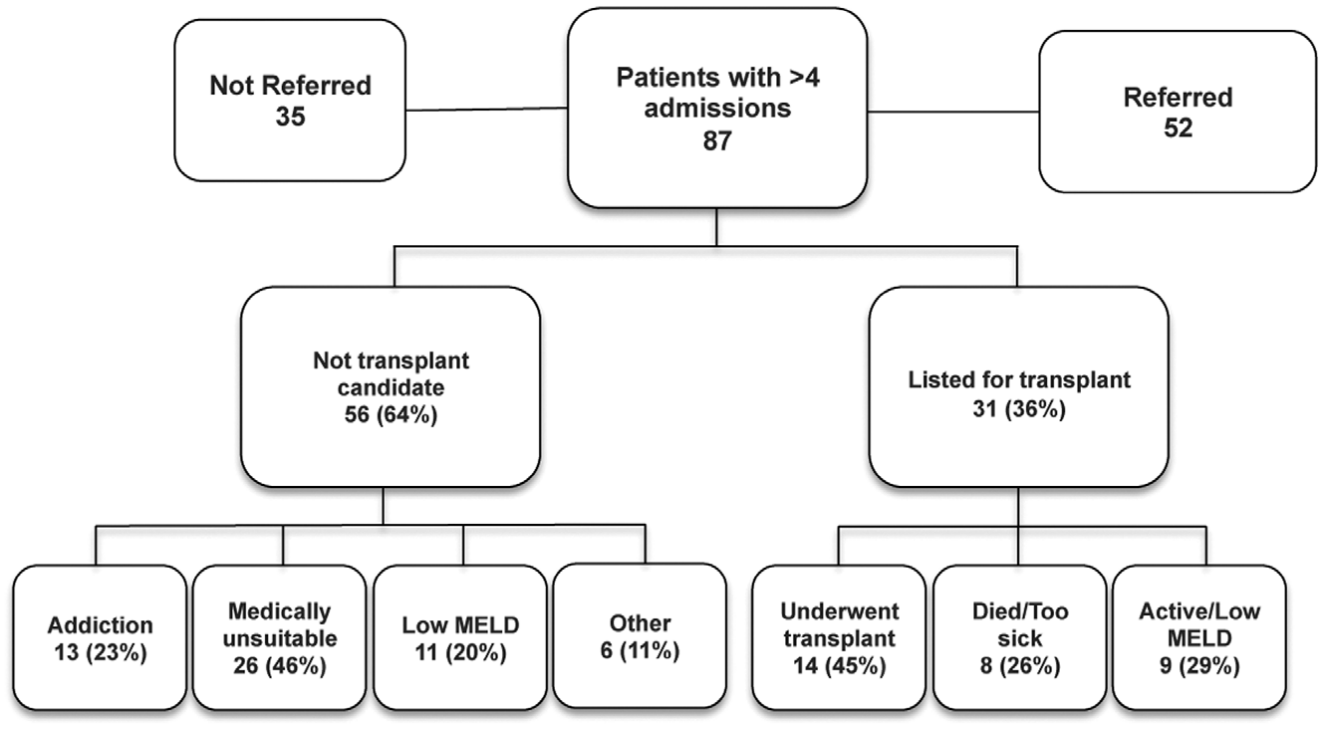
Flowchart showing transplant-related outcomes in patients with >4 admissions. Patients deemed to not to be transplant candidates include both referred and non-referred patients. Low MELD score patients had MELD <15.

Since liver transplantation is an important indication for patients with decompensated cirrhosis, we determined potential barriers to transplantation for patients in the high-readmission group (Figure 2). Just 14 (16%) patients in this group underwent liver transplantation during the study period and an additional 9 (10%) patients were active on the transplant list, including 6 patients that had MELD score <15. Eight (9%) patients were listed for transplant but either died (5 patients) or inactivated from the transplant list due to acute illness or deconditioning (3 patients). The mean time for all patients between referral to listing was 204±247 days, with a median of 111 days. The 9 patients that were both referred and listed for transplant in the study period had an average of 1.3 (range 0–3) admissions between referral and listing.

Fifty-six (64%) of 87 patients in the high-readmission group were deemed unsuitable candidates for transplantation. Nine of these patients died during the study period. Barriers to transplantation in this group of patients included active alcohol and drug use with or without psychiatric co morbidities in 13 (15%) patients, low MELD score (<15) in 11 (20%) patients, and medical comorbidities, advanced age, inoperable hepatocellular carcinoma, or extra hepatic cancers in the remainder. Two patients were not listed for transplant due to lack of health insurance.

## Discussion

As the debate over healthcare costs intensifies and the issue of hospital readmission rates gets increased regulatory scrutiny, it is important to identify patients who may benefit from targeted interventions to decrease the risk of readmissions. Our study makes three important contributions to the field. First, to the best of our knowledge, our study is the first to show that in a cohort of patients (cirrhotics) at high risk of hospitalization, a relatively small group accounts for a disproportionate number of readmissions. Second, we demonstrate that racial disparities may be contributing to the risk of readmissions in patients with cirrhosis. We also corroborate previous studies that MELD score and diabetes are factors increasing risk of readmission in patients with cirrhosis. Third, we establish that cirrhosis patients with frequent readmission have significant barriers to transplantation that could potentially be targeted to increase access to transplantation and mitigate the high readmission rates.

Recent studies demonstrate that early re-hospitalizations among patients with decompensated cirrhosis are common, with 20–37% of patients being readmitted within 1 month of discharge [7], [8]. Early readmission was associated with MELD score, diabetes, and male gender, while the time to readmission in another study was predicted by MELD score, serum sodium level, and number of medications on discharge [8]. In patients with hepatitis C-related cirrhosis, readmission rate at 1-year was 45% with a remarkably high admission rate of 73% among patients with hepatic encephalopathy [12]. Taken together, these studies strongly suggest that the prevention of hospital readmissions in patients with end-stage liver disease represents a major unmet healthcare need deserving of the same level of attention from the healthcare community that has been accorded to patients with chronic obstructive pulmonary disease or congestive heart failure. Chronic disease management is now a widely accepted to address chronic disease outcomes in patients with heart disease and diabetes [13]. However, while inconsistency of care of cirrhosis has been well-recognized and quality indicators have been put forth for cirrhosis care [14], chronic disease management in cirrhotics has not been well studied or established. The goal of our study was to identify cirrhosis patients at high risk of frequent hospital admissions and identify potential barriers to liver transplantation.

That patients with decompensated cirrhosis have high readmission rates would by itself not be surprising. However, a significant finding of our study was that a relatively small number of patients (not necessarily the medically sickest patients as assessed by conventional scoring criteria) accounted for a disproportionately large percentage of hospital admissions in this population. We found that MELD score was a strong predictor of frequent hospital admissions, a finding that is consistent with prior reports and with the MELD score as being a validated predictor of prognosis in patients with cirrhosis. In addition, race and diabetes were associated with frequent admissions. Prior studies have shown that African-Americans having the highest rates of preventable hospitalization for congestive heart failure, diabetes, and hypertension [15]–[17]. Whether these findings are also true for patients with cirrhosis will require further research, including controlling for potential confounders of educational and socioeconomic status [15], [18]–[21]. Diabetes alone does not explain the racial disparity in readmission; given that race was still a factor despite controlling for diabetes. Given the increased risk of frequent admissions in patients with diabetes, our results raise the question whether better control of diabetes could be a potential intervention in this population to decrease hospitalization. Prior studies have shown that diabetes is also a risk factor for hepatic encephalopathy and may have contributed to frequent hospitalizations in these patients [22], [23]. Our study has implications for clinical practice for healthcare providers as well for hospital administrators, policy makers, and health insurance companies who make decisions regarding allocation of resources towards the care of these patients.

Since liver transplantation is now considered an important therapeutic option in patients with advanced liver disease, we were interested in determining potential barriers to transplantation in the group of patients at the highest risk of admissions. Not surprisingly, we found that this group had significant barriers to transplantation, including active alcohol use and addiction issues. Thus, our results raise the possibility that targeting this group for early and aggressive intervention directed at addiction and psychiatric comorbidities may be an option to decrease hospitalizations and increase access to transplantation. Interestingly, several patients in this group, despite their obviously advanced liver disease, had low MELD scores (<15). Since MELD score of great than 15 is required in the US for deceased donor transplantation, this subgroup represents unique challenges to manage their disease. Thus, it is intriguing to speculate whether interventions such as early transjugular intrahepatic portosystemic shunt placement in patients with cirrhosis, special considerations in the organ allocation system, or live donor liver transplant in patients with ascites would decrease the frequency of admissions in this group of patients and provide overall more cost-effective care [24]–[26]. Our study identifies groups of patients who are ineligible for liver transplant and at high risk for admissions towards which specific, personalized interventions can be targeted to minimize risk of hospitalization. Indeed, insights gained from our study has led to changes in practice patterns at our institution, including early referral to drug and alcohol treatment programs, aggressive management of fluid status in patients identified on their index admission to be at risk of readmission, and more timely referrals to palliative care.

Some limitations of our study should be considered. We did not have data on education or socioeconomic status to control for potential confounders in terms of the differences we saw in terms of race. Since the goal our study was to discover risk factors for multiple readmissions, time-based analyses could not be used. Therefore, we controlled for non-uniform follow-up interval by including follow-up time in the model. In order to minimize the differences in follow-up time, patients with less than 90 days of follow-up were excluded from the study. Excluding these patients could potentially lead to bias, therefore, we also analyzed the data by including the group with shorter follow-up times and the predictive factors remained the same and just 3 additional patients were included in the hyper-admission group. The choice of 5 admissions for the cut-off was based on the both on the mean number of admissions per person year as well as the fact that this group accounted for half of the total admissions in the study group. These results are of particular interest because we show that targeting this small group of patients and optimizing their transplant status could potentially decrease the risk of half of all admissions.

In summary, a relatively small number of patients with end-stage liver disease account for a disproportionately large number of hospital admissions. Diabetes, race, and MELD score were predictive of high risk of admissions. In this group of patients at the highest risk of admissions significant barriers to transplant exist. Interventions designed to address these factors, including low MELD score despite having decompensated cirrhosis and addiction issues could potentially increase access to transplantation and decrease frequency of hospitalizations.
